# Laparoscopic Adrenalectomy: Tailoring Approaches for the Optimal Resection of Adrenal Tumors

**DOI:** 10.3390/diagnostics13213351

**Published:** 2023-10-31

**Authors:** Ionela Mihai, Adrian Boicean, Cosmin Adrian Teodoru, Nicolae Grigore, Gabriela Mariana Iancu, Horatiu Dura, Dan Georgian Bratu, Mihai Dan Roman, Cosmin Ioan Mohor, Samuel Bogdan Todor, Cristian Ichim, Ioana Bogdan Mătacuță, Ciprian Băcilă, Nicolae Bacalbașa, Ciprian Nicolae Bolca, Adrian Hașegan

**Affiliations:** 1 Faculty of Medicine, Lucian Blaga University of Sibiu, 550169 Sibiu, Romania; ionela.mihai@ulbsibiu.ro (I.M.); adrian.boicean@ulbsibiu.ro (A.B.); adrian.teodoru@ulbsibiu.ro (C.A.T.); nicolae.grigore@ulbsibiu.ro (N.G.); gabriela.iancu@ulbsibiu.ro (G.M.I.); horatiu.dura@ulbsibiu.ro (H.D.); mihai.roman@ulbsibiu.ro (M.D.R.); cosmin.mohor@ulbsibiu.ro (C.I.M.); samuelbogdant@gmail.com (S.B.T.); cristian.ichim@ulbsibiu.ro (C.I.); ioana.matacutabogdan@ulbsibiu.ro (I.B.M.); ciprian.bacila@ulbsibiu.ro (C.B.); adrian.hasegan@ulbsibiu.ro (A.H.); 2 Faculty of Medicine, University of Medicine and Pharmacy “Carol Davila”, 020021 Bucharest, Romania; nicolaebacalbasa@gmail.com; 3 Surgery Department, Universite de Sherbrooke, Sherbrooke, QC J1K 2R1, Canada; ciprian.nicolae.bolca@usherbrooke.ca

**Keywords:** laparoscopic adrenalectomy, adrenal tumors, transperitoneal approach, retroperitoneal approach

## Abstract

In this study, we investigated the outcomes of laparoscopic approaches for adrenal tumor resection in 67 patients from a single center with a median age of 51 (range 40–79). Predominantly comprising women, the majority of patients were overweight or obese. Adrenal tumors larger than 6 cm were mostly treated using the laparoscopic transperitoneal method (*p* < 0.001). Our results revealed that patients subjected to the retroperitoneal approach exhibited quicker recovery, as evidenced by faster resumption of oral intake and ambulation, along with reduced intraoperative blood loss and shorter hospitalization (*p*-value < 0.05). In contrast, patients subjected to the transperitoneal approach experienced minimal complications, though not statistically significant, despite the technique’s intricacy and slower recovery. These findings emphasize the significance of tailoring the surgical approach to individual patient characteristics, with particular emphasis on the tumor size. The choice between the retroperitoneal and transperitoneal methods should be informed by patient-specific attributes to optimize surgical outcomes. This study underscores the need for a comprehensive evaluation of factors such as tumor characteristics and postoperative recovery when determining the most suitable laparoscopic approach for adrenal tumor resection. Ultimately, the pursuit of individualized treatment strategies will contribute to improved patient outcomes in adrenal tumor surgery.

## 1. Introduction

Tumors in the adrenal glands represent a broad spectrum of pathological entities, covering a diverse array of conditions. These tumors span from benign non-hormone-secreting adrenocortical adenomas to hyperfunctional malignancies, like adrenocortical carcinoma, and hormone-secreting adrenal medullary tumors, such as phaeochromocytoma [1]. While many adrenal tumors receive medical attention due to symptoms, often centered around hypertension, incidental discovery also plays a significant role [2]. This is exemplified by the identification of adrenal enlargement during abdominal imaging conducted for unrelated purposes [3].

An “adrenal incidentaloma” denotes an adrenal tumor exceeding 1 cm in size, serendipitously unveiled during the course of imaging acquisitions. This phenomenon has gained prominence within clinical spheres and owing to the escalating volume of radiological investigations conducted, leading to its heightened prevalence in current medical practice [4]. The majority of adrenal incidentalomas manifest as benign and hormonally quiescent adrenal adenomas. Nonetheless, it is imperative to accurately diagnose and effectively manage potentially life-threatening entities like adrenal carcinoma and functional tumors [4,5,6].

At times, the possibility and identification of an adrenal gland tumor might emerge in situations involving a family history or a medical syndrome associated with inherited genetic tendencies. Particularly notable among these are neurofibromatosis type 1, endocrine neoplasias types MEN 1 and MEN 2, genetic alterations affecting succinate dehydrogenase (SDH) and Von Hippel–Lindau syndrome (VHL) [7,8].

Surgery remains a crucial treatment approach, often standing as the primary option in certain specific cases. The key factor driving the need for adrenalectomy constitutes the presence of a tumor that releases hormones, resulting in excessive hormone release and related symptoms, or a confirmed/suspected diagnosis of malignancy [9]. Adrenalectomy involves the precise surgical removal of the adrenal gland. When it comes to treating adrenal tumor pathologies, laparoscopic surgery is the preferred treatment option. This technique has witnessed remarkable evolution since its inception through the pioneering work of Gagner in 1992 [10]. There has been a significant shift in the approach toward adrenal diseases, from the conventional approach to laparoscopy, both for malignant as well as benign conditions. Numerous studies demonstrate the benefits of laparoscopic adrenal surgery, including less blood loss, fewer hospital days, quicker recovery and fewer complications than traditional open surgery [6,9].

Over the course of time, numerous laparoscopic methods have been documented. Notably, the anterior and lateral transperitoneal approaches, alongside the lateral and posterior retroperitoneal approaches, have emerged as prominently employed methodologies [11]. Additional methods have been recently introduced, although their widespread adoption is still limited. These include robot-assisted surgery, single-port surgery and hand-assisted laparoscopy [12,13,14,15].

The lateral transperitoneal approach is well-suited to removing tumors with diameters ranging from 8 to 15 cm. This method provides improved exposure due to gravity’s influence and benefits from the surgeons’ familiarity with the anatomical structures [16,17,18].

Patients who have undergone prior abdominal surgeries may find the posterior retroperitoneal approach beneficial because it does not require adhesions to be broken down. As a result, no other abdominal organs are involved in this technique, which allows direct access to the adrenal gland. This approach also allows the surgeon to remove tumors from both adrenal glands without necessitating patient repositioning. This approach is suitable for dealing with larger tumors between 6 and 10 cm in size, although it might not be the best choice for individuals with a body mass index exceeding 40 kg/m^2^ [15,16,19,20].

Lately, the scope of laparoscopic surgery has been broadened, encompassing indications that now include metastatic lesions and malignant tumors, all while maintaining a focus on surgical safety and addressing to cases that involve pheochromocytoma [21]. The selection of the approach hinges on the surgical team’s preferences and expertise, in addition to any existing contraindications, which might be influenced by the patient’s positioning during the procedure [4,9].

## 2. Material and Methods

In a retrospective analysis, we examined 82 patients with adrenal tumor pathologies who underwent laparoscopic adrenalectomy via both retroperitoneal and transperitoneal approaches within the Urology Department of Sibiu County Clinical Hospital over the past 8 years.

The surgical procedures were carried out by the same surgical team. Among these, 67 were enrolled in the study, with the remaining cases not meeting the selection criteria. The inclusion criteria comprised patients aged 18 and above, those with comprehensive medical histories and complete blood analyses, individuals undergoing laparoscopic interventions and cases with histopathological examinations confirming adrenal tumor pathology. Patients with diagnostic uncertainties or those not subsequently followed up at our center were excluded from the study.

In addition to assessing each patient’s hormonal profile before surgery, abdominal CT or MRI was used to diagnose tumors and identify potential malignancies. A multidisciplinary team assessed each individual’s state of health, leading to the establishment of the surgical intervention necessity.

Every patient provided informed consent, acknowledging the proposed surgical intervention while being fully aware of alternative treatment options and comprehending the potential intraoperative and postoperative risks.

Before surgery, treatment options were meticulously assessed, considering clinical and histopathological attributes, tumor location, origin (primary or metastatic), expected postoperative recovery duration and potential complications associated with each specific surgical approach. Patients with active functional tumors received tailored preoperative treatment and biochemical adjustments, all under the care of the same surgical team. The favored surgical techniques encompassed both the retroperitoneal and transperitoneal laparoscopic methods.

The functional status of the adrenal tumors was determined by means of the dosage of urinary catecholamines, cortisol and ACTH tests, and hormonal tests showing the presence of pheochromocytoma and Cushing’s syndrome.

In statistical analysis, categorical variables were presented as a count (percentage %) and compared using Fisher’s exact test, while continuous variables were presented as a median (Interquartile range) and compared using the Kruskal–Wallis test. Normality was assessed using the Shapiro–Wilk test. The threshold of the *p*-value was set at 0.05 2 sig.tailed. IBM SPSS version 22.0 was used for statistical analysis.

## 3. Surgical Technique Specifics

As previously noted, the imaging assessment predominantly relied on computer tomography scans for the majority of patients, while MRI and positron emission tomography were employed in only two cases. For a precise diagnosis, both CT and MRI imaging were conducted in seven patients.

**Figure 1:**
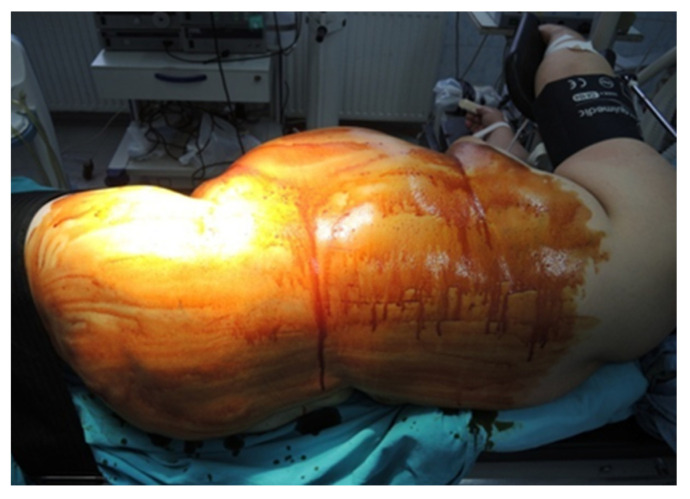
Position in the retroperitoneal lateral approach (personal collection).

**Figure 2:**
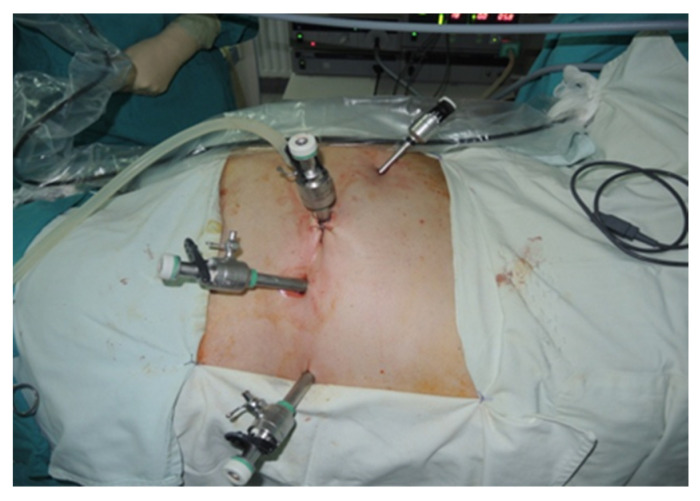
Positioning of trocars (personal collection).

During the lateral retroperitoneal approach, the patient is placed in a lateral decubitus position (as illustrated in Figure 1). The primary surgeon and the assisting medical professional on the surgical side stand behind the patient, with the second assistant positioned in front. At the uppermost point of the 12th rib, a 1.5 cm incision is made to access the retroperitoneal space. Subsequently removing the subcutaneous tissue with scissors, the retroperitoneal fascia is meticulously traversed, followed by digital dissection to establish the operative workspace. Trocars are then introduced under either digital or visual guidance (a total of four trocars, as depicted in Figure 2). The psoas muscle, ureter, diaphragm and kidney’s posterior aspect are notable anatomical landmarks within the retroperitoneal space.

When the working space is created for the removal of the left adrenal gland, the Gerota’s fascia is carefully cut at the upper renal pole level and then carefully maneuvered towards the medial surface. Before cutting the adrenal gland, hem-o-lok clips are used to secure the adrenal gland’s central vein. The vascular circle is subjected to electrocoagulation to ensure the thorough mobilization of the gland. The extracted gland is enclosed within a specialized organ bag, either by widening a trocar incision or utilizing two adjoining trocar incisions, depending on the gland’s size.

Right adrenalectomy entails locating the right renal and the inferior cava vein at the intersection of the central adrenal vein, which originates cranially on the lateral side, post Gerota’s fascia incision. This adrenal vein is ligated using hemostatic clips before being divided. This procedure follows the same steps as the left side.

**Figure 3:**
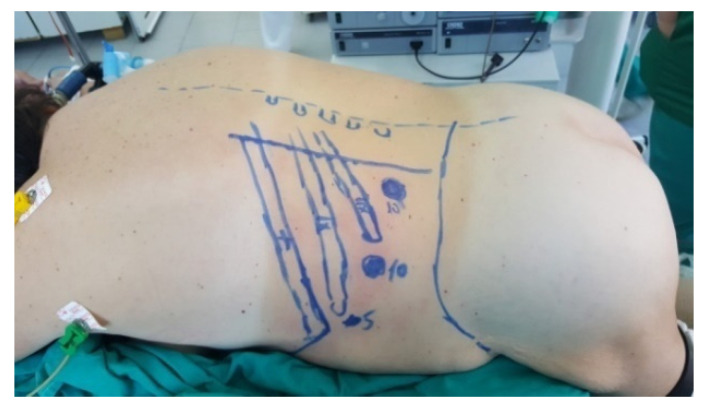
Position in the retroperitoneal posterior approach (personal collection).

**Figure 4:**
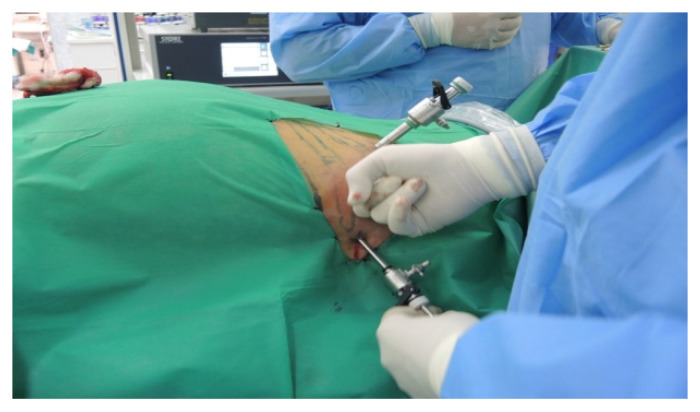
Positioning of trocars (personal collection).

The patient is placed in a ventral decubitus position with the legs propped up on a support to facilitate a relaxed abdominal wall in the posterior retroperitoneal approach. This positioning strategy aids in expanding the retroperitoneal space (as illustrated in Figure 3). The pathway to the retroperitoneal space is established at the 12th rib level via a 1.5 cm incision. This incision is followed by a process of digital dissection to establish the operative workspace. An 11th rib thoracotomy and a costomuscular angle thoracotomy are performed with trocars placed under digital guidance (a total of three trocars, as shown in Figure 4).

Anti-Trendelenburg angling and flexing of the operating table allow the patient to be placed in a lateral position, 45 degrees from horizontal, in the transperitoneal approach. This positioning maximizes the gap between the 12th rib and the iliac crest. The surgical region encompasses the space between the pubic symphysis and the base of the xiphoid appendix, extending outwards towards the posterior axillary line. The surgeon and assistant stand in front of the patient’s abdomen, with the surgeon on the side of the tumor, and the assistant on the opposite side.

Creating the pneumoperitoneum involves inserting the Veress needle at the navel level, passing it through the supraumbilical fold and entering the peritoneal cavity. The CO_2_ insufflator is then connected. At 15 mmHg, the working trocars are introduced, followed by the Veress needle being removed and a 12 mm optical trocar being inserted. This trocar allows the peritoneal cavity to be laparoscopically inspected and subsequent trocars to be introduced under visual guidance, using the same technique as in partial or radical nephrectomy. In order to assist liver retraction, a 5 mm subxiphoid trocar is needed for the right adrenal gland.

In the case of left-sided suprarenalectomy, the procedure entails the following steps: after parietal peritoneum incision and division of the freno-colic ligament, the left renal vein is located. Before cutting the central vein, the upper aspect of the vein is marked and secured with hem-o-lok hemostatic clips. This initial stage is pivotal, as it effectively halts the release of adrenal gland secretion products caused by manipulation during surgery. Subsequent phases involve incising the Gerota’s fascia, locating the adrenal gland and proceeding with dissection between the aorta and the gland. The arterial pedicles are clipped, enabling the gland’s mobilization towards the diaphragm. Hem-o-lok hemostatic clips are used to ligate the inferior phrenic arterial pedicle and its branch, the superior adrenal artery. The extracted gland is enclosed within an organ bag through a trocar-incision combination or a trocar-widened incision, contingent on its size.

**Figure 5:**
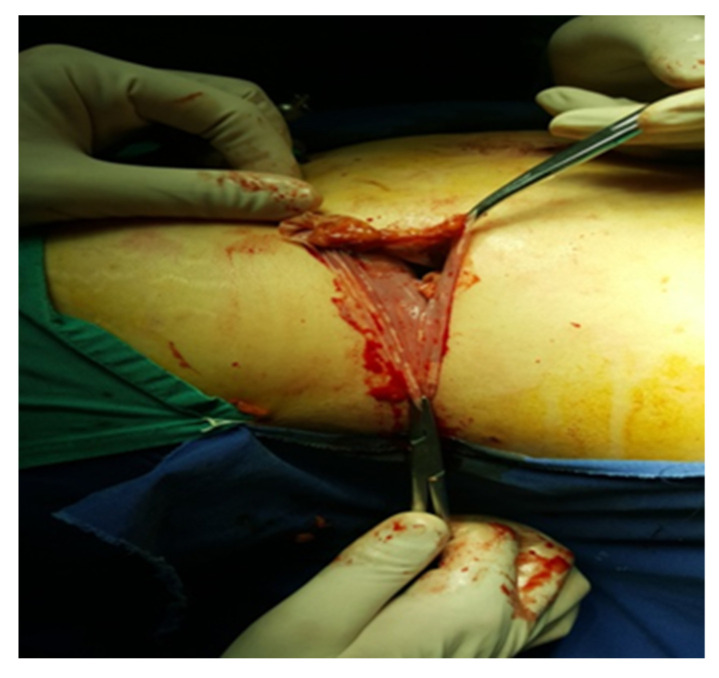
Excision of the piece (personal collection).

An incision is made along the hepatic flexure of the colon from the level of the duodenum and inferior vena cava for right-sided adrenalectomy. In addition to locating and fastening the central adrenal vein with Hem-o-Lok hemostatic clips, the inferior vena cava and duodenum are separated from the cranial segment of the inferior vena cava. Subsequent dissection of the gland involves the clipping or electrocoagulation of adrenal arteries, followed by the extraction of the gland into the organ bag (Figure 5).

Intraoperatively, the CO_2_ levels are closely monitored through capnogram readings and arterial Astrup measurements taken from the radial artery. In cases of pheochromocytoma, a strategy is employed to avert the discharge of catecholamines induced by cold exposure. To achieve this, prewarmed CO_2_ is administered to these patients.

**Figure 6:**
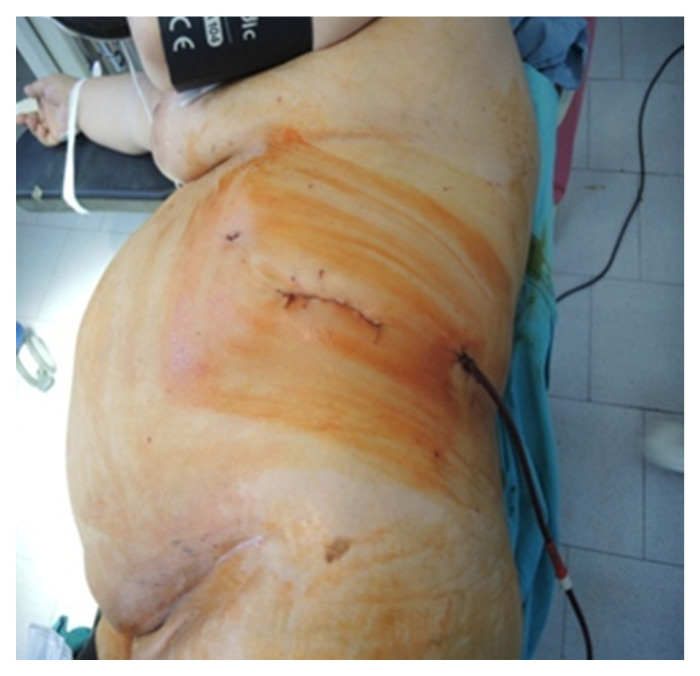
Final aspect of the lateral approach (personal collection).

**Figure 7:**
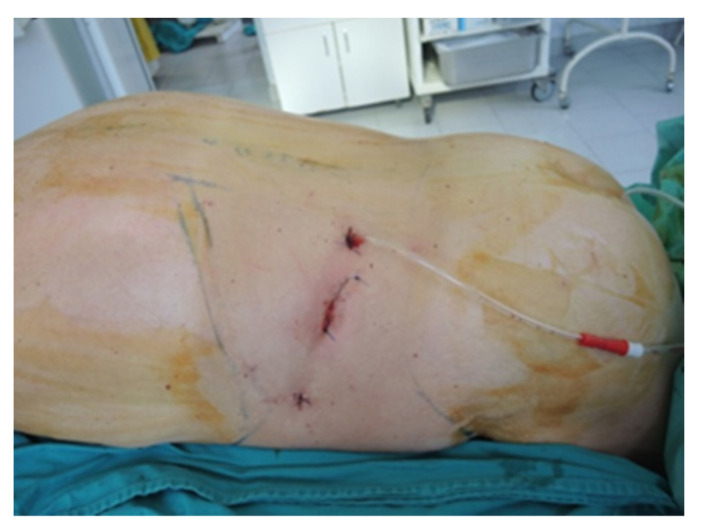
Final aspect of the ventral approach (personal collection).

**Figure 8:**
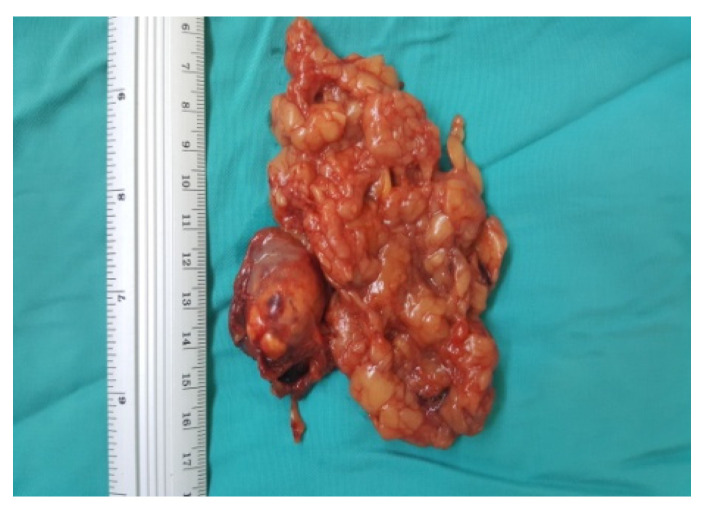
Surgical specimen (personal collection).

Final aspects of the surgery are depicted in Figure 6, Figure 7 and Figure 8. 

## 4. Results

In all cases, laparoscopic surgery was performed without converting to open surgery. In patients operated on via the retroperitoneal approach, mobilization and intestinal transit were resumed on the first postoperative day and on the third day for patients operated on via transperitoneal approach. Adrenal function normalized in all cases of pheochromocytoma with normalization of blood pressure, without notable events of postoperative blood glucose values.

In the subset of patients who underwent adrenalectomy, a female gender was more common (61.2%). The median age of this cohort study was 51 years, with ages ranging from 40 to 79 years. The body mass index (BMI) distribution reflected the following categories: overweight (BMI 25–30)—18 individuals; obese (BMI 30–40)—24 individuals; and morbidly obese (BMI > 40)—5 individuals.

Concerning the laparoscopic techniques utilized, the methods can be categorized as follows: the anterior transperitoneal approach was used in 12 cases, the lateral retroperitoneal approach was utilized in 31 cases and the posterior retroperitoneal approach was selected in 24 cases (Table 1).

Within the studied cohort, the median diameter of tumors was recorded at 5.4 cm (ranging from 3.1 to 10.2 cm), with the majority of cases (67.17%, 45 cases) exhibiting tumors measuring less than 6 cm in diameter. A noteworthy proportion of these tumors were situated on the left adrenal gland, constituting 36 cases (53.7%).

In terms of surgical methodologies, the laparoscopic retroperitoneal approach was the dominant choice, being employed in the majority of cases, totaling 55 patients. The posterior retroperitoneal approach was selected for 24 patients (35.8%), the lateral retroperitoneal approach was selected for 31 patients (46.2%), and in 12 patients (18%), the transperitoneal approach was utilized.

Primary adrenal gland tumors were identified in a substantial portion of the cases, totaling 59 instances (88.1%). Of these, 49 cases (73.1%) underwent excision via the retroperitoneal approach, a notably higher percentage than the 14.92% observed for the transperitoneal approach.

Secondary tumors were detected in nine patients, with the majority of them being managed through the retroperitoneal approach (six cases, 8.95%). This is in sharp contrast to the patients who underwent the transperitoneal approach. The sources of these secondary tumors were primarily pulmonary neoplasia (n = 4), followed by liver cancer (n = 2), retroperitoneal tumors (n = 2), and melanoma (n = 1).

The highest blood loss during procedures was recorded in cases where the transperitoneal approach was employed, with a median value of 155 mL (140–164.3). In terms of intervention duration, the transperitoneal laparoscopic approach proved to be the most laborious, with a prolonged median intervention time (Table 2). Moreover, patients undergoing the latter intervention require more post-operative analgesia, have a late resumption of oral intake and ambulation, and extended hospitalization.

A singular case of *Clostridioides difficile* was encountered. However, it was deemed independent of the surgical approach, as all patients received antibiotic therapy.

## 5. Discussions

Laparoscopic adrenalectomy stands out as the most efficient and expedient approach for addressing adrenal gland-related issues. When performed by a skilled surgeon, it becomes the preferred technique for treating adrenal pathologies. Nevertheless, the ideal approach remains a subject of debate. An internationally recognized pattern does not currently exist, and further studies are required to establish a consensus within the medical community.

Even though most tumor types (except for gender-related tumors, i.e., ovarian tumors) are more frequent in men, a review showed that adrenal tumors are more frequent in female patients (incidentalomas, oncocytomas, adrenal cysts and adrenal carcinomas). Moreover, female patients more often report signs of pheochromocytomas [22].

Due to the complexity of the surgical procedure for adrenalectomy, the preferred anesthesia approach involves a combination of general anesthesia and epidural anesthesia. This choice is based on its relatively lower associated risks and the additional benefit of improved pain management throughout both the intraoperative and postoperative periods, with the epidural catheter providing a distinct advantage [23]. All patients receive antibiotic therapy in such interventions, so the surgeon and anesthetist must always be aware of the risk of antibiotic resistance (a significant public health issue) and *Clostridioides difficile* infection, which can be prevented in many situations. If the case does not respond to conventional treatment, microbiota transplantation remains a viable option [24,25,26,27].

### 5.1. Robotic Surgery vs. Traditional Laparoscopy

It is undeniable that the landscape of surgery has undergone a remarkable transformation over time, with robotic procedures increasingly making their presence felt, particularly in the realm of urology. One interesting question is whether robotic surgery will eventually become the dominant method, leaving traditional laparoscopy in the past. Over the past two decades, the scientific community has produced an extensive body of literature, offering insights into the efficacy and potential pitfalls of robotic surgical techniques. One crucial preliminary step in embracing this approach is establishing the comprehensive safety profile of robotic surgery in addressing a wide spectrum of adrenal tumor cases [28]. As is the case with any medical intervention, it is essential to weigh the advantages against the disadvantages. Numerous research studies have undertaken the task of comparing classical laparoscopy with this innovative method, carefully evaluating a set of surgical criteria, as delineated in one of the tables provided earlier. These criteria encompass variables such as operating times, the volume of blood loss, the duration of patient hospitalization, as well as the incidence of complications, both intraoperative and postoperative.

In their comprehensive research efforts, Ozer Makay and their colleagues arrive at a resounding conclusion: robotic surgery delivers superior outcomes, demonstrating a marked reduction in blood loss and shorter hospital stays for the patients undergoing such procedures. An intriguing revelation within their findings is that the rates of both intraoperative and postoperative complications show no significant disparities when compared to conventional laparoscopy [29].

Importantly, another pivotal study in the literature, involving a substantial cohort of over 1000 patients, supports the observation of reduced hospitalization durations while emphasizing the decreased occurrence of complications in the context of laparoscopic adrenalectomy. Furthermore, within a more detailed study, Micaella P. and colleagues propose that the future may indeed see a role for robotic adrenalectomy, particularly in highly selective and challenging cases. These may include patients with obesity and those with notably large adrenal tumors. Additionally, the advantages of robotic surgery become evident in cases involving tumors located on the right side of the body [30].

In order to further mitigate risks and complications, modern technology can be of significant assistance, regardless of whether adrenalectomy is conducted through laparoscopy or with the use of robotics. The progression of technology has led to the development of systems based on artificial intelligence, which can provide several essential functions [31].

Firstly, these systems can offer rapid and precise imaging diagnosis of adrenal pathologies. They excel at swiftly and accurately identifying various adrenal conditions, classifying them, and providing a comprehensive understanding of the nature of the issue. This early diagnostic capability can greatly aid in preoperative assessment and planning.

Secondly, these advanced AI-based systems can provide real-time assistance to the surgeon during the surgical procedure. They play a crucial role in helping the surgeon to identify and understand the intricate vascular anatomy in real-time. This feature is invaluable in ensuring that the procedure is performed with a high degree of precision and safety [32].

### 5.2. Transperitoneal vs. Retroperitoneal Approach

Selecting the optimal surgical approach remains a pivotal step in urological surgery. Regardless of the chosen method, it is important to mention that in right-sided surgery, both the transperitoneal and retroperitoneal approaches have demonstrated therapeutic safety.

However, in other cases, there are subtle differences between the methods, so numerous factors should be considered, ranging from the surgeon’s expertise, patient’s weight and comorbidities to the potential risks of complications. From a specialized literature standpoint, complications stemming from such interventions can be manifold, owing to the critical organs that may be affected during the operative procedure [33]. In this study, the transperitoneal surgical approach exhibited the fewest post-operative complications, significantly outperforming other types of interventions.

Within the studied group, individuals subjected to the retroperitoneal approach experienced complications, such as wound infections. These outcomes stand in contradiction to certain literature findings, which fail to identify significant safety discrepancies between the two surgical approaches [34]. In cases involving interventions on small-sized tumors, there is a unanimous consensus that a transperitoneal surgical procedures should be avoided or approached with exceptional caution [35].

Obesity continues to be an increasingly prevalent risk factor in everyday practice. As observed in Inaishi’s study [20], obesity did not yield any additional complications among this group of patients, regardless of the surgical approach. However, cases with a high BMI pose technical challenges and extend surgical times, involving difficult and less secure manipulation of instruments. Consequently, there exists the potential for a considerable rise in complications or mortality within a larger patient cohort. Therefore, as advocated by numerous publications, the transperitoneal approach remains the preferred choice for individuals with a BMI ≥ 30 kg/m^2^. This is due to its simplicity, providing enhanced visualization of abdominal organ anatomy and a spacious operative field conducive to resecting larger tumors [36,37,38,39].

Over a century ago, Mercan et al. pioneered laparoscopic retroperitoneal adrenalectomy [40], and since then, it has gained widespread use as a surgical technique for adrenal tumor resection. However, even after a span of 30 years, there is still no consensus regarding the ideal approach from all perspectives. Recent significant studies, such as that conducted by Alberto Arezzo et al., have not yielded conclusive results for most of the comparative aspects in the context of the retroperitoneal and transperitoneal approaches [41].

In contrast to other esteemed authors, our study identified statistically significant differences between the two types of approaches for adrenalectomy [42]. Specifically, the transperitoneal approach exhibited significantly greater blood loss and prolonged operative time. This outcome is reinforced by the anatomical organization of the body. Retroperitoneal surgery has been found to have a shorter surgical duration and lower average bleeding because of the placement of the adrenal glands within the retroperitoneal space, as well as the direct approach. This happened due to the minimal intra-abdominal dissection required compared with the transperitoneal method. The adrenal gland is accessed directly in the retroperitoneal approach, eliminating the necessity to maneuver the liver, pancreas and spleen. Consequently, this results in a reduced risk of additional injuries, bleeding, and procedural elongation. The resumption of intestinal transit and the recovery period were significantly quicker with the retroperitoneal approach, a fact that aligns with the findings of Mohammad Reza Mohammadi-Fallah et al. [43].

Table 3 presents several of the most esteemed clinical research findings in the existing literature, conducted to compare the effectiveness of different adrenalectomy approaches. Despite the age of these studies, the lack of result uniformity remains noteworthy, particularly the subsequent contradictions. These disparities have carried over into subsequent review and meta-analysis studies as well.

Zhang et al. [49] advocated for and established retroperitoneoscopic lateral adrenalectomy as the predominant approach for addressing adrenal tumors in China. Consequently, surgeons have the option to consider various approaches, including lateral, posterior and anterior approaches, when conducting laparoscopic suprarenal gland removal [50,51]. The surgeon’s expertise and the choice of materials can exert a substantial impact on the case’s progression, leading to a significant reduction in interventional risks [52,53]. Professionals must continuously stay abreast of the latest findings in their field of expertise based on the specialized literature.

Regarding the method of resection—total or partial adrenalectomy—studies have shown that both have a similar safety profile, and in primary adrenal neoplasias, the outcomes were similar in terms of progression [54,55]. Beyond this aspect, numerous scoring systems have been created with the aim of predicting the long-term outcome of adrenalectomy. Among these, perhaps the most accurate score was presented in the study by Umberto Anceschi et al., with the new trifecta system being able to evaluate the long-term outcome in both partial and total adrenalectomy [56].

## 6. Conclusions

By combining findings from literature studies with the results obtained from our center, several conclusions can be drawn. However, these will necessitate confirmation through multicenter studies involving a substantial number of patients.

The retroperitoneoscopic approach (lateral or posterior) to adrenal tumor resection has multiple advantages: a minimal dissection space, clearly defined anatomical landmarks, avoiding the pneumoperitoneum and contact of the intestines with blood, rapid postoperative recovery, and superior aesthetic appearance.

We advocate for a individual approach regarding laparoscopic adrenal tumor resection, taking into account all the possible risks. One of the main deciding factors could be the tumor size.

Even though the transperitoneal approach involves a more laborious technique and slower recovery, translating into later resumption of oral intake and ambulation and a prolonged hospital stay, it was found to be beneficial and was associated with minimal complications when adopted for the resection of large adrenal tumors.

New studies have the potential to enhance the literature, not only through detailed comparisons between the lateral and posterior retroperitoneal approaches, but also between laparoscopic surgery and niche surgeries such as single-port and robot-assisted procedures.

## Figures and Tables

**Table 1:** General characteristics of the patients.

Variable	P.R.L.	L.R.L.	T.L.	Total	*p*-Value
	24 (35.8%)	31 (46.2%)	12 (18%)	67 (100%)	
Sex					
Female	18 (26.9%)	16 (23.9%)	7 (10.4%)	41 (61.2%)	0.221
Male	6 (8.9%)	15 (22.4%)	5 (7.5%)	26 (38.8%)	0.121
BMI (IQR)	30.5 (26–32.3)	31.8 (27.3–33.2)	30.9 (20–28)	30.7 (26–33.2)	0.093
Position					
Left adrenal gland	15 (62.5%)	13 (41.9%)	8 (66.6%)	36	0.087
Right adrenal gland	9 (37.5%)	18 (58.1%)	4 (33.3%)	31	0.137
Dimensions					
Average diameter (cm)	4.0 (3.1–6.4)	4.2 (3.1–7.2)	7.5 (5.8–10.2)	5.4 (3.4–8.4)	0.023
Under 6 cm	21 (87.5%)	22 (70.1%)	2 (16.6%)	45	<0.001
Over 6 cm	3 (12.5%)	9 (29.9%)	10 (83.4%)	22	<0.001
Specific details					
Primary tumor	20 (83.3%)	29 (93.5%)	10 (83.3%)	59	0.437
Secondary tumor	4 (16.6%)	2 (6.5%)	2 (16.6%)	8	0.437
Incidentaloma	8 (33.3%)	7 (22.5%)	2 (16.6%)	17	0.493
Sdr. Cushing	1 (4.1%)	2 (6.4%)	1 (8.3%)	4	0.873
Pheohcromocytoma	4 (16.6%)	5 (16.2%)	1 (8.3%)	10	0.777
Adenoma	10 (41.6%)	13 (41.9%)	6 (50%)	29	0.874
Primary carcinoma	1 (4.1%)	1 (3.2%)	2 (16.6%)	4	0.223
Cyst	0	3 (9.6)	0	3	0.325

All of the variables are expressed as counts (%) and compared using Fisher’s exact test. BMI is presented as a median (IQR) and compared using the Kruskal–Wallis test. *p* < 0.05; P.R.L., posterior retroperitoneal laparoscopic; L.R.L., lateral retroperitoneal laparoscopic; T.L., transperitoneal laparoscopic.

**Table 2:** Characteristics of the procedure type and post-operative monitoring.

	Posterior Retroperitoneal	Lateral Retroperitoneal	Transperitoneal	*p* Value
Number of cases	24	31	12	
Duration of the intervention (min)	86.5 (52.3–110.3)	85.2 (53.6–115.3)	115 (60.1–153) *	<0.05
Blood loss (average mL)	65.9 (60–78)	66.7 (61.2–82)	155 (140–164.3) *	<0.05
Resumption of food postoperatively (h)	7.6 (3.2–40)	8.1 (4–41)	20.4 (5–88) *	<0.05
Resumption of mobility (h)	5.7 (3–20.4)	6.1 (4.1–22)	22 (7–36) *	<0.05
Average length of hospitalization (days)	3.52 (1–5)	3.67 (1–5)	4.62 (3–7) *	<0.05
Postoperative analgesia (h)	1.7 (1–4)	2.2 (1–4)	3.1 (1–5) *	<0.05
Conversion to open surgery	2 (8.3%)	2 (6.4%)	1 (8.3%)	0.958
Postoperative complications	1 (4.1%)	2 (6.4%)	0 (0%)	0.543

* Statistically significant differences are flagged and indicated through significant pairwise comparisons.

**Table 3:** Common findings in the literature.

Study	No. of Patients	Approach Type	Results
Rubinstein et al. [44]	57	Transperitoneal and retroperitoneal	No notable disparities in surgical duration, blood loss, hospitalization duration, or complications. Differences in convalescence time
Berber et al. [45]	172	Transperitoneal and retroperitoneal	Higher blood loss and hospitalization rate in those operated by transperitoneal approach
Lee et al. [46]	43	Transperitoneal and retroperitoneal	Shorter operative time and shorter time to resumption of feeding in retroperitoneal approach
Ramacciato et al. [47]	171	Transperitoneal and retroperitoneal	Lower operating time and blood loss in transperitoneal approach; Shorter time to resumption of feeding in retroperitoneal approach
Chai et al. [48]	48	Transperitoneal and posterior Retroperitoneoscopic	Less blood loss and reduced surgical duration in the retroperitoneal method

## Data Availability

The datasets generated and analyzed during the current study are not publicly available due to institutional restrictions, but are available from the corresponding author upon reasonable request.
